# The Dark Triad of Particulate Matter, Oxidative Stress and Coronary Artery Disease: What About the Antioxidant Therapeutic Potential

**DOI:** 10.3390/antiox14050572

**Published:** 2025-05-09

**Authors:** Daniele Grifoni, Elisa Bustaffa, Laura Sabatino, Francesca Calastrini, Fabrizio Minichilli, Melania Gaggini, Sergio Berti, Cristina Vassalle

**Affiliations:** 1 Institute of Bioeconomy (IBE), National Research Council (CNR), Via Madonna del Piano 10, 50019 Sesto Fiorentino, Italy; grifoni@lamma.toscana.it (D.G.); calastrini@lamma.toscana.it (F.C.); 2 Laboratory of Monitoring and Environmental Modelling for the Sustainable Development (LaMMA Consortium), Via Madonna del Piano 10, 50019 Sesto Fiorentino, Italy; 3 Institute of Clinical Physiology, National Research Council, Via G. Moruzzi 1, 56124 Pisa, Italy; elisa.bustaffa@cnr.it (E.B.); laura.sabatino@cnr.it (L.S.); fabrizio.minichilli@cnr.it (F.M.); melania.gaggini@cnr.it (M.G.); 4 Fondazione CNR-Regione Toscana G Monasterio, Ospedale del Cuore “Gaetano Pasquinucci”, Via Aurelia Sud, 54100 Massa, Italy; berti@ftgm.it; 5 Fondazione CNR-Regione Toscana G Monasterio, Via G. Moruzzi 1, 56124 Pisa, Italy

**Keywords:** oxidative stress, particulate matter, cardiovascular disease, acute myocardial infarction, health, antioxidants, PM_2.5_

## Abstract

Particulate matter (PM) is a complex mixture of particles with different adverse effects on health, especially on the cardiovascular (CV) risk and disease (e.g., increased risk of total and CV mortality, ischemic heart disease, heart failure, stroke, hypertension, dyslipidemia and type 2 diabetes). Since oxidative stress (OS) and inflammation are the main key mechanisms by which PM exerted its biological effects on health, several oxidative and inflammatory-related biomarkers have been measured and associated with PM; abnormalities in these parameters in relation to PM highlight the key role of this relationship in terms of adverse health effects, including CV conditions. Antioxidant strategies might prevent/reverse, almost partly, CV effects related to PM exposure, by addressing OS and inflammation, although the clinical gain of these interventional tools is not yet clearly demonstrated. This review aims to summarize PM source and composition, discussing OS and inflammatory events associated with environmental PM exposure as key mechanistic determinants of CV risk and acute event precipitation. Moreover, the modifying potential of antioxidants, especially in subjects more susceptible to the adverse effects of air pollution and/or more highly exposed, will be discussed as a promising research area beyond conventional strategies actually available to prevent the harmful effects of PM (e.g., reduction of pollution sources and population exposure, assessment of air quality standards) in order to better face this dark triad composed of PM, OS and CV disease.

## 1. Introduction

Air pollution is a complex combination of gaseous and particulate molecules, which greatly changes according to geographical, urban/rural, meteorological characteristics and many other factors [1,2]. Particulate matter (e.g., PM_10_, which includes particles < 10 µm in diameter, PM_2.5_ for particles < 2.5 µm) and gases such as nitric oxide (NO), nitrogen dioxide (NO_2_), sulfur dioxide (SO_2_) and ozone (O_3_), are among the primary environmental threats to human health and well-being, affecting multiple organ systems [2,3]. In particular, an increase in mortality from exposure to PM, especially PM_2.5_, has been evidenced since the 1990s [4]. Accordingly, exposure to air pollution is now considered a significant contributor to global health risks: it is among the top five risk factors, out of eighty-seven, in a global assessment, alongside other major health concerns such as an unhealthy diet and tobacco smoking [5]. The World Health Organization (WHO) estimates that approximately 4 to 9 million deaths annually are related to air pollution [6,7] and the Global Burden of Disease study attributes 4.1 million of these deaths to ambient PM_2.5_ [5]. Thus, the majority of clinical studies have generally focused on acute and chronic exposure to PM (with stronger evidence for PM_2.5_, judged the most dangerous air pollution component among the first cause of mortality in 2015); in this context, the WHO (2021) has set limits, recommending that the annual average PM_2.5_ should not exceed 5 µg/m^3^, whereas daily levels should remain below 15 µg/m^3^ [7,8,9].

At present, there is a strong case for causality between pollution and a wide range of cardiovascular (CV) endpoints [10]. In agree with its importance in terms of CV effects, air pollution has been recognized as a key determinant for CV disease (CVD), and one of the modifiable factors which can be modulated in the prevention and management of CVD by an expert position paper on air pollution and CVD from the ESC Working Group on Thrombosis, which declared: “There is now abundant evidence that air pollution contributes to the risk of cardiovascular disease and associated mortality, underpinned by credible evidence of multiple mechanisms that may drive this association” and “air pollution should be viewed as one of several major modifiable risk factors in the prevention and management of cardiovascular disease” [11]. This relationship is particularly evident for PM_2.5_, as also summarized by the American Heart Association “evidence is consistent with a causal relationship between PM_2.5_ exposure and cardiovascular morbidity and mortality”, which also reported that the majority of deaths (57–76%) attributable to PM_2.5_ are a result of atherosclerotic events, supporting a consistent relationship between both short-term (days to weeks) and chronic (years) air pollution exposure and the occurrence of acute myocardial infarction (AMI) and stroke; nonetheless, increasing evidences are emerging also on the role that the gaseous component might have in terms of CV pathophysiology [12,13]. In fact, the CV system is one of the major health targets of PM, not only due to CVD’s high prevalence as the leading cause of morbidity and mortality, but also because the CV system facilitates the systemic distribution of pollutants, exacerbating damage in multiple organs. Accordingly, the risk functions for PM_2.5_ and CVD, showing a relative risk of 1.11, with a 95% confidence interval (CI) ranging from 1.09 to 1.14, were also updated by WHO in 2021 [7].

It is known that PM_2.5_ promotes inflammation, oxidative stress (OS) and a hypercoagulable state, all of which act as key determinants in the relationship between this pollutant and CVD. In this context, growing evidence suggests that antioxidant intake might prevent/reverse, almost partly, CV effects related to PM exposure, counteracting the increase of OS and inflammation, although the clinical gain of these interventional strategies is not yet clearly demonstrated. Thus, this review aims to summarize PM source and composition, with a focus on OS and inflammatory events elicited by environmental PM exposure as key mechanistic culprits of CV risk and acute event precipitation. It also explores the potential for antioxidants in modifying these adverse effects on CV pathophysiology.

## 2. Search Strategy

We utilized the PubMed search to evaluate the available topic-related studies published in English on the subject of “oxidative stress effects of air pollution on the CV system” to discuss in this narrative review. Studies specifically focusing on cancer, cerebrovascular or respiratory pathologies were excluded. Instead, special focus was placed on PM effects, because its concentration is generally found closely related to CV morbidity and mortality.

Different combinations of following main search terms were used: “antioxidants”, “oxidative stress”, “inflammatory disease”, “air pollution”, “particulate matter” or “PM”, “vehicle emission”, “cardiovascular”, “CVD”, “blood pressure”, “cardiac”, “coronary heart disease”, “ischemic heart disease”, “short term”, “long term” and “mortality”. Then, articles relevant to our topic were selected based on the title, abstract and full text of each paper.

## 3. Particulate Matter

Atmospheric aerosol, or PM, consists of a broad class of solid or liquid particles with different chemical and physical properties. Aerosol is therefore a heterogeneous collection of particles that differ not only in their chemical and physical properties, but also in their formation mechanisms or emission sources, which can be either natural or anthropogenic. The main natural sources are mineral desert dust, sea spray, volcanic ash, forest fires and pollen, while the main anthropogenic sources are linked to the use of fossil fuels for energy production, heating, land, air and sea transport, as well as to industrial, mining and even agricultural activities. Aerosol can be emitted directly from the source as primary PM or produced in the atmosphere as secondary PM through chemical reactions between primary solid, liquid and gaseous pollutants. Studying aerosols is therefore challenging, as they are a collection of heterogeneous particles that can vary during time because of chemical and physical processes, and in space as a result of transport, dilution and deposition processes [14,15].

The effects on the environment, climate and human health are also closely related to the characteristics and transformation processes of PM itself. Since the 1970s, numerous studies in various scientific fields, including climatology, biogeochemistry and medicine, have emphasized the significant impact of atmospheric aerosols, from the global to the urban scale and even the indoor environment [16,17,18,19,20]. These studies have highlighted the need to monitor this pollutant with standardized measurement systems and specific analyses. In this context, various methodologies have been proposed for the classification of PM based on characteristics such as size, chemical composition, formation processes and origin. The purpose of these classifications is to better understand the environmental and health impacts associated with these properties [15,21,22,23].

### 3.1. Particle Size Fractions

One of the main classification methodologies is based on particle size, as this influences radiation scattering and absorption, atmospheric visibility and human health [23,24,25]. The parameter used to express the size of particles is the equivalent aerodynamic diameter (D_a_), defined as the diameter of a spherical particle with a density of 1 g/cm^3^ and a settling velocity as that of the particle in question under the same conditions of temperature, relative humidity and pressure [15]. This definition is necessary because, unlike liquid particles that tend to assume spherical shape, solid particles generally have irregular shapes. The D_a_ can vary significantly from the physical diameter, with variations influenced by factors such as density and aerodynamic properties, which depend on the particle’s surface area and volume.

In the classification of particles by size, three different methods are used: modal, based on the observed size distributions and formation mechanisms; sampler cut point, which refers to the particle diameter at which a sampling device collects a certain percentage (commonly 50%) of particles of that size; dosimetry, based on the particle penetration into different compartments of the human respiratory system [15,23].

#### 3.1.1. Modal Classification

**Figure 1:**
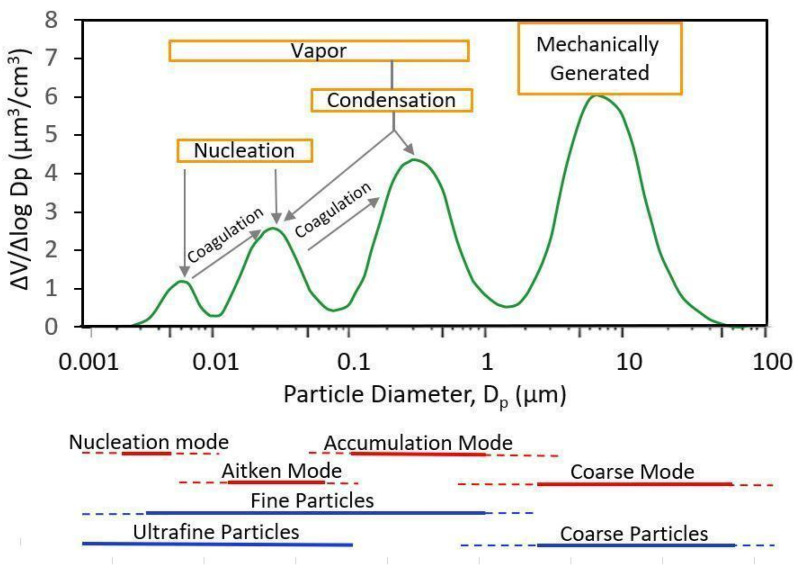
An idealized size distribution, which might be observed in traffic, showing fine and coarse particles, as well as the nucleation, Aitken and accumulation modes (adapted from [15]).

Particle size and formation processes are closely linked: the modal classification, by considering the aerosol distribution as a function of particle diameter, allows the identification of classes of particles formed through different chemical and physical processes [14,15,23]. The idealized aerosol modal distribution shown in Figure 1 highlights four modal peaks: coarse, accumulation, Aitken and nucleation mode.

Coarse Mode—The coarse mode consists of particles with diameters that exceed the minimum in the particle mass distribution (the point in the size range of particles where the mass of the aerosol particles is at its lowest), which typically occurs between 1 and 3 µm. The formation of such particles is predominantly due to mechanical processes (e.g., soil erosion, industrial activities such as mining and construction), resuspension (e.g., by wind or human activity such as vehicle traffic) or biogenic sources (such as pollen or spores). This fraction includes dust from desert or arid regions, sea salt from the ocean, and also nitrate and sulfate formed from chemical reactions of nitric acid with sodium chloride and SO_2_ with basic particles, respectively. The coarse mode and the accumulation mode overlap in the region between 1 and 3 µm. The sedimentation rate of the particles is faster compared to other modes, such that they can be deposited within hours or days.

Accumulation Mode—The particles with diameters from about 0.1 µm to 1–3 µm are mainly derived by coagulation (smaller particles collide and combine to form larger ones), by condensation (low-equilibrium vapor pressure gas molecules condense onto existing particles or nucleate to form new particles). Additionally, these particles may result from the fragmentation of larger particles. The mechanisms for removing this fraction are less effective, so the residence time in the atmosphere is in the order of days or longer.

Aitken Mode—This mode is constituted by particles with diameters ranging from 0.01 to 0.1 µm. This mode is also referred to as the *transient nucleation mode*. It is the result of particles formed by nucleation (a process by which gaseous substances condense or react to form new solid particles or liquid droplets), as well as by condensation and coagulation.

Nucleation Mode—Freshly formed particles observed during active nucleation events with diameters below about 0.01 µm. The nucleation mode can be observed as a separate mode in clean or remote areas or near sources. The residence time in the atmosphere for this fraction, as for the Aitken mode, is short, on the order of hours.

The term “Fine particles Mode” refers to the fraction of particles with diameters smaller than the minimum in the particle mass distribution, between 1 and 3 µm. The fine mode includes the accumulation, the Aitken and the nucleation modes. Fine particles are formed primarily by combustion or chemical reactions of gases (e.g., industrial emissions, vehicle exhaust emissions). They are composed of sulfate, nitrate, ammonium and hydrogen ions, metals and metal oxides, black carbon or elemental carbon and organic compounds (OCs). Elemental carbon is directly emitted into the atmosphere mainly from combustion processes, whereas OCs can have both primary and secondary origins through the condensation of products in the process of hydrocarbon photooxidation. The secondary component of OCs is a considerable fraction of the total OC [14].

The ultrafine particles (UFPs) are generally defined by size, as particles with diameters of 0.1 µm or less. While UFP includes the nucleation mode and much of the Aitken mode, they are not considered a mode in themselves. The effects of UFP on light scattering and absorption are relevant, producing visibility alterations. Furthermore, these particles act as cloud condensation nuclei. Their ability to penetrate deep into the lungs, enter the bloodstream and induce OS makes them a significant concern for air quality regulations and public health [15].

#### 3.1.2. Sampler Cut Point Classification

To measure size fractions of particles relevant to health, environment, etc., size-selective sampling has been developed, i.e., the collection of particles below or within a specified aerodynamic size range. For example, dichotomous samplers split the particles into two distinct size fractions: smaller and larger, while cascade impactors separate airborne particles into multiple size fractions. A variety of upper-size cut samplers with a single filter are also used. The term cut point is used to describe the performance of particle-size selective devices. Typically, the maximum size of particles that the device will collect with 50% efficiency is defined by the upper 50% cut point size [23].

**Figure 2:**
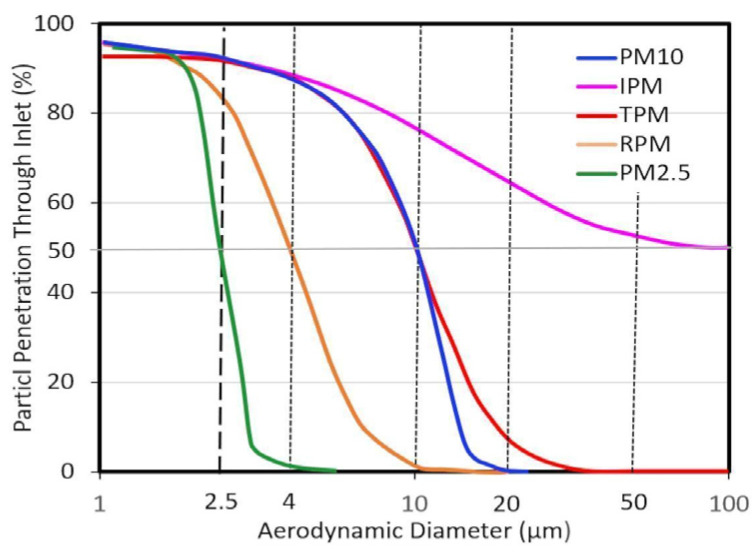
Specified particle penetration (size-cut curves) through an ideal inlet for five different size-selective sampling criteria. Regulatory size cuts are defined in the Code of Federal Regulations: PM_2.5_ [27], PM_10_ [26]. Size-cut curves for inhalable particulate matter (IPM), thoracic particulate matter (TPM) and respirable particulate matter (RPM) size cuts are defined by the ACGIH [29] (adapted from [15]).

The use of PM_10_, the aerosol fraction with a D_a_ less than 10 µm [26], and PM_2.5_, the fraction with a D_a_ less than 2.5 µm [27], as indicators is an example of size-selective sampling based on a legally defined, regulatory size for air quality standards. It is important to note that PM_10_ and PM_2.5_ are formally recognized as regulated pollutants in international and European legislation [28]. The threshold choice of PM_10_ is based on health considerations, as particles of this size are able to enter the thoracic compartment (see Figure 2). Conversely, the threshold choice of PM_2.5_ is driven by its distinct sources, rather than its respirable fraction. Numerous studies have demonstrated that the two fractions differ in chemical and physical characteristics, toxicity, emission sources, etc. [14,15,17,22,23].

#### 3.1.3. Dosimetry Classification

Since 1993, standardized classification of airborne particles based on their ability to penetrate the human respiratory system has been adopted by the American Conference of Governmental Industrial Hygienists [29], the International Standards Organization and the European Committee for Standardization. These organizations established three key fractions of PM: inhalable, thoracic and respirable particles, according to their upper size cut-offs [15,23,29].

Inhalable fraction: these particles enter the respiratory tract through the nose and mouth. Thoracic fraction: these particles pass beyond the larynx and reach the tracheo-bronchial region. Respirable fraction: this fraction represents a subset of the thoracic particles that are small enough to penetrate even further, reaching the alveolar region of the lungs. In addition, UFP can pass through the alveolar barrier and enter the bloodstream.

Based on dosimetry classification, the curves defining inhalable particulate matter, thoracic particulate matter and respirable particulate matter are shown in Figure 2. This classification ensures accurate evaluation of how different particle sizes affect respiratory health, providing a framework for air quality limits and occupational exposure regulations [29].

### 3.2. Main Sources and Composition of Particulate Matter

The sources of atmospheric aerosols can be either anthropogenic or natural. Anthropogenic PM originates in urban or industrial areas, primarily from traffic emissions (both exhaust and non-exhaust), domestic heating, construction activities and various industrial activities (such as power plants, oil refineries, mining, etc.). In rural areas, major sources are represented by agricultural activities, including biomass burning. Natural aerosols mainly consist of soil and desert dust, sea spray, volcanic ash, emissions from vegetation and wildfires. These different sources, through a range of chemical and physical processes, generate particles with different chemical compositions and varying environmental and health impacts [14,17,22].

#### 3.2.1. Traffic

Vehicular traffic, especially in urban areas, is an important source of both primary and secondary aerosols. Particle sizes vary depending on the formation processes: vehicles emit a mixture of ultrafine primary carbon particles [30] and gases, including NO_2_, a precursor of nitrogen compounds, as part of exhaust emissions [31], while non-exhaust emissions result from abrasion due to tyre wear, brake wear, road wear and the resuspension of road dust [32]). Brake and tyre wear releases metals in small concentrations such as copper, zinc and cadmium [33], while traces of other elements, including potassium, bromine and chlorine, originate from the engine. PM emissions from diesel vehicles are typically higher than those from gasoline vehicles and contain toxic chemicals such as polycyclic aromatic hydrocarbons (PAHs), which are linked to adverse health effects [34].

Maritime transport is a major contributor to air pollution in terms of SO_2_ emissions and sulfate aerosols. It is also responsible for NO_2_ emissions and carbonaceous PM, with a considerable contribution on a global scale [35,36].

Similarly, aviation plays a role in aerosol and precursor gas emissions. Aircraft engines release metals such as aluminum, titanium, chromium, iron, nickel and barium [37,38].

#### 3.2.2. Industrial Activities

Emissions from the industrial sector are heterogeneous as they derive from a variety of industries, including those of petrochemical, metallurgical, ceramic and pharmaceutical sectors. Consequently, these emissions are influenced by different production cycles and raw materials used. The activities with the most significant impact are power plants, oil refineries and mining. Energy production from fossil fuels is a major contributor to the emission of secondary aerosol precursor gases. Additionally, coal combustion generates primary PM as well as sulfur, carbonates, chlorides and metals such as mercury [39], while oil combustion releases sulfates and metals such as vanadium and nickel, which can be used as tracers specific for these sources [40].

Industrial activities related to the production of ceramics, bricks and cement, as well as foundries and mining, are significant contributors to primary PM emissions. These activities also release production-specific heavy metals. For instance, the metallurgical industry emits metals such as copper, iron, manganese and zinc, which are used as markers for source identification [41,42].

#### 3.2.3. Biomass Burning

Biomass burning is also an important source of aerosols and gases that contribute to increased air pollution and climate-changing emissions, at both regional and global scales. This source includes forest fires, which can be natural or, more often, human-induced for purposes such as pastoral or agricultural land use. The burning of agricultural biomass residues in fields to prepare the land for the next planting season also has a relevant impact on air quality worldwide [43,44,45].

Wood burning for domestic heating is a significant source of atmospheric pollution due to biomass combustion. In winter, domestic biomass burning has been identified as a relevant source of pollution not only in rural areas, but also in residential areas of Europe [46,47,48] and the USA [49]. In Europe, more than 40% of fine PM emissions are attributed to domestic combustion [50]. Many studies have shown that stable atmospheric conditions together with low temperatures, which encourage the use of domestic heating, can lead to critical air quality conditions in the Po Valley (Northern Italy) [51] and Tuscany (Central Italy), where domestic heating is a major contributor to PM_10_ concentrations during the cold season [52,53,54,55].

Emissions from wood burning depend on combustion conditions and the characteristics of the biomass, such as the species of wood used [56]. The main components of the aerosol emitted from biomass burning are carbonaceous compounds, including OC and elemental carbon [57]. Elements that are produced during the decomposition of cellulose, such as levoglucosan, can be used as markers of this source [55]. The inorganic component of the emitted aerosol contains mainly potassium, ammonium, sulfate and nitrate [57]. Interestingly, recent data indicate the importance of wood burning, which significantly increases fibrinogen in cardiac patients and contributes to an increased risk of AMI in elderly subjects [58,59].

#### 3.2.4. Mineral Desert Dust

Mineral desert dust is a major component of atmospheric aerosols on a global scale [20,60,61]. They play a crucial role in cloud formation and radiative transfer [62,63,64], influence both terrestrial and oceanic ecosystems, and affect air quality by reducing visibility and impacting human health [65,66,67,68].

The sources are mainly dried or ephemeral lakes or rivers, located within the desert or semi-arid regions of the subtropics. The most important of these is the Sahara, but other major sources are in Arabia, Central Asia, the southwestern United States and Australia [20,69,70]. Mineral dust is mainly composed of calcite, quartz, dolomite, kaolinite, illite, feldspar and traces of calcium sulfate and iron oxides [71], although the chemical and mineralogical composition differs by region of origin [72]. Europe, and particularly the Mediterranean region, is also frequently affected by desert intrusions, which are more frequent in spring and summer. In some cases, high desert dust concentration can contribute to the exceedance of legal PM_10_ concentration limits [73].

In recent years, the frequency and intensity of desert intrusions in the Mediterranean area have increased [74,75]. These intense episodes, characterized by very high PM_10_ concentrations, have been associated with adverse effects on human health. Recent studies also indicate an increase in mortality from CVD associated with these events [65].

#### 3.2.5. Sea Spray

Sea spray, or marine aerosol, is one of the largest contributors to atmospheric aerosol worldwide [76]. While most prevalent in coastal areas, sea spray can also be found inland [77]. This type of aerosol is mostly primary and consists mainly of sodium and chloride, with smaller amounts of other components such as sulfate, potassium, magnesium and calcium. In addition, sea spray has a secondary component mainly consisting of OC produced by phytoplankton. Among these, dimethyl sulfide, one of the main precursors of atmospheric sulfates in oceanic regions [78].

#### 3.2.6. Biogenic Emissions

Biogenic emissions, produced by vegetation and microorganisms, contribute to the formation of primary and secondary atmospheric aerosols. Primary particles include pollen, spores, but also bacteria, viruses, proteins and carbohydrates [79]. Biogenic volatile OC, e.g., isoprene and terpenes, can act as precursors of secondary aerosol [14,80]. In addition, by interacting with anthropogenic emissions (volatile organic compounds and nitrogen oxides-NOx), biogenic volatile organic compounds contribute to the formation of tropospheric O_3_ [81].

## 4. Particulate Matter and Cardiovascular Diseases

Many studies have shown that long-term exposure to PM_2.5_ is correlated to subclinical atherosclerosis (AS; increase in coronary artery calcium (CAC) or carotid intima-media thickness (IMT)), heart failure (HF) and CV mortality [82]. Recently, de Bont and colleagues performed an umbrella review summarizing the current epidemiological evidence from systematic reviews and meta-analyses linking ambient air pollution (PM and NOx) to multiple CVD manifestations [83]. Specifically, sufficient evidence was observed for the following: (i) both short- and long-term exposure to PM and increased risk of CVD mortality, ischemic heart disease and AMI, (ii) short-term PM exposure and increased risk of stroke, blood pressure, HF and arrhythmias, (iii) short-term NOx exposure and increased risk of stroke and arrhythmias and (iv) long-term NOx exposure and an increased risk of ischemic heart disease and AMI [83]. These associations may be affected by different factors, such as by AMI subtypes (e.g., ST-elevation myocardial infarction-STEMI and non-ST-elevation myocardial infarction-NSTEMI, according to different characteristics of electrocardiogram-ECG), lag times and individual specificities and susceptibility (e.g., elderly subjects or presence of comorbidities as type 2 diabetes-T2D). Moreover, the effects of pollutants are evident not only in more vulnerable groups (e.g., those with comorbidities but also those with asthma, and children) but even in healthy subjects [84,85]. Accordingly, PM_2.5_ short-term exposure is able to induce ECG changes also in healthy subjects [86].

Long-term joint exposure to air pollutants, including PM_2.5_, PM_10_, NO_2_ and NOx, has been repeatedly found to be associated with increased risk of subclinical AS. An association between IMT and PM_10_ mass concentration was observed in 2348 subjects living in London [87]. A meta-analysis including eight cohorts (*n* = 18,349) for the assessment of cross-sectional association between IMT and PM exposure and three cohorts (*n* = 7268) for the longitudinal analysis on carotid IMT and PM, confirmed this relationship [88]. A systematic review including eighteen studies (five cohorts and thirteen cross-sectional), also supported the existence of a positive association between PM exposure and subclinical AS (CAC and IMT) [89]. In a prospective Chinese study (*n* = 8867 aged 25–92 years with suspected coronary artery disease—CAD), a significant association between long-term exposures to PM_2.5_ and NO_2_ with an increase in CAC scores was observed [90]. Accordingly, in a German cohort (4814 middle-aged adults, 5 years follow-up) long-term exposure to PM_2.5_ was found associated to development and progression of subclinical AS (1.5 μg/m^3^ higher exposure to PM_2.5_) with an odds ratio of 1.19 (95% CI: 1.03, 1.39) for progression of CAC, with an increased annual growth rate of 2% (95% CI: 1%, 4%) [91].

Moreover, in the Swedish CArdioPulmonary bioImage Study Gothenburg study (2013–2017, *n* = 5070, age 50–64 years), although no consistent relation between long-term total PM_2.5_ exposure and CAC score or presence of carotid artery plaques was observed, an association between total PM_2.5_ and larger plaque area in participants with carotid plaques was found; positive correlation with traffic-related air pollutants were found for both a high CAC score and bilateral carotid artery plaques [92]. These associations were stronger among men and those with CV risk factors [92]. In fact, PM may act as an endocrine disruptor and, as such, interfere with hormones (e.g., insulin), contributing to the onset and progression of metabolic diseases (e.g., obesity and T2D) [93]. Moreover, PM_2.5_ and PM_10_ have been associated with an increased risk of developing hypertension and impairment of high-density lipoprotein (HDL) function [94,95].

However, in the Malmö Diet and Cancer study between 1991 and 1994 (*n* = 6103), no clear relationship was found between air pollution exposure and carotid plaque prevalence [96], whereas the existence of controversial data reflects the complexity and difficulty of studying the relationship between pollutants and CV pathophysiology, which depends on many determinants. Nonetheless, different meta-analyses support the relation between long-term exposure to air pollutants and HF incidence, and other outcomes (e.g., hospital readmissions) [97,98,99,100,101], while other meta-analyses highlight the association between overall and CV mortality and air pollutants [102,103].

Interestingly, satellite-based estimates of long-term PM_2.5_ exposure were associated with both CAD and AMI incidence in cardiac catheterization patients; in particular, 1 μg/m^3^ increase in annual average PM_2.5_ gave an 11.1% relative increase in the odds of CAD (95% CI: 4.0–18.6%) and a 14.2% increase in the odds of having an AMI within a year prior (95% CI: 3.7–25.8%) [104]. A meta-analysis (27 cohort studies, 6,764,987 participants and 94,540 AMI patients) reported that higher levels of PM_2.5_ and PM_10_ exposure were significantly associated with AMI risk (relative risk for each 10 μg/m^3^ increment in PM_2.5_ and PM_10_ corresponding 1.18 (95% CI: 1.11–1.26) and 1.03 (95% CI: 1.00–1.05), respectively) [105].

Different studies also confirmed a significant link between short-term exposure to pollutants (e.g., PM_10_ and PM_2.5_) and the rate of hospitalizations for CV events (especially the occurrence of HF and AMI) [84,85], finding a 2% increased risk of AMI with each 10 μg/m^3^ exposure to PM_2.5_ (relative risk of 1.02; 95% CI, 1.01–1.03; *p*-value ≤ 0.0001) [85]. In agreement with this observation, short-term exposure to PM_2.5_ and PM_10_ was associated with increased triggering of both mortality and hospital admissions for AMI. Additionally, an increase in long-term exposure to PM_2.5_ was linked to a higher risk of AMI mortality/incidence [83].

A case-crossover study design (*n* = 12,865, USA) evidenced the relationship between PM_2.5_ and acute ischemic coronary events (unstable angina and AMI; 10 µg/m^3^ elevation associated with increased risk of 4.5%, 95% CI, 1.1–8.0) [106]. Recent data showed that, in areas with a long-term moderate or high severity of air pollution, short-term exposure to high concentrations of PM_2.5_ and PM_10_ (10 μg/m^3^ increase) is positively correlated with both AMI and acute HF [107].

An Italian study performed in winter, when PM_2.5_ are characterized by the presence of nitrate, organic carbon fraction, with high amount of PAHs and elements such as lead, aluminum, zinc, vanadium, iron, chromium and others, evidenced the potential of PM_2.5_ to alter global gene expression in heart tissue (181 upregulated and 178 downregulated genes; e.g., increase in collagen and laminin related genes as well as in genes involved in calcium signaling), highlighting the question of individual susceptibility, and the need to protect more vulnerable subjects [108].

The heterogeneous composition of PM—differing by source, region and season—impacts its toxicity. In this context, the disparate effects of traffic-related PM (enriched in metals and PAHs) have been more studied compared to desert dust (rich in silicates) effects, which remain little investigated.

Experimental and in vitro studies suggested that desert dust is correlated with OS and inflammation, mitochondrial dysfunction, an increase in mean blood pressure and heart rate, as well as being associated with increased blood pressure in humans [109,110,111,112].

A systematic review with meta-analysis (20 cohort studies), investigating the effects of occupational silica exposure on the risk of heart disease, evidenced that silica-exposed workers are at a higher risk for overall heart disease, with stronger evidence supporting an association with pulmonary heart disease [113]. Moreover, a Japanese study on 3068 consecutive AMI patients showed that exposure to desert dust a few days before symptom onset is associated with the incidence of AMI [114]. Interestingly, more recent data provide evidence that short-term exposure to desert dust is associated with a higher risk of a particular type of myocardial infarction with nonobstructive coronary arteries (MINOCA), compared to myocardial infarction with CAD [115].

These results open new opportunities for future studies; if there is still much to understand, a better knowledge of how the heterogeneous composition of PM has differential effects of toxicity may be helpful to guide targeted public health strategies informed by PM source characteristics and in accordance with the relevance to various exposure scenarios.

## 5. Oxidative Stress and Other Mechanisms Mediating Particulate Matter Effects on the Cardiovascular System

Main pathways involved in eliciting the adverse CV outcomes due to pollutant exposure may be broadly categorized into the following:

(1) Primary initiating responses in the lung—these occur following pollutant inhalation and include (a) either exogenous (pollutant-induced) or endogenous OS, (b) pulmonary inflammation and (c) ion channel/receptor activation;
(2) Transmission pathways—these facilitate the systemic impact of initial pulmonary responses and include (a) generation of biologic intermediates (e.g., oxidized lipids, cytokines, activated immune cells, microparticles, microRNA, vasoconstrictors) (b) autonomic imbalance/afferent neurological circuits leading to the central nervous system (sympathetic or hypothalamic pituitary adrenal axis activation);
(3) End-organ effector mechanisms—the previous pathways, in turn, lead to end-organ effector mechanisms responsible for atherosclerotic events [116].

Several biomarkers have been identified in the association between PM and CAD, whereas many others are probably not discovered or fully elucidated, mainly involving endothelial function, OS and inflammation, but also dyslipidemia, increased thrombogenicity and blood pressure. In this context, endogenous antioxidants could act as defense mechanisms that can reduce the stimulation of these particles, modifying the relationship between OS and air pollutants. Main endogenous antioxidants include glutathione (antioxidant and xenobiotic detoxifier), uric acid (a major blood antioxidant), bilirubin (with antioxidant and anti-inflammatory activity), catalase (one key antioxidant enzyme) and superoxide dismutase (SOD) (detoxification enzyme and powerful antioxidant).

Different interindividual capacities to modulate the antioxidant response may be a reason for different susceptibility to pollutant damage. Consequently, air pollution induces erythrocyte enzyme inactivation (Glutathione peroxidase 1 (GPx-1) and Cu, Zn-SOD), although some subjects showed positive GPx-1 and Cu, Zn-SOD correlation with air pollutants. This response could be related to a greater capacity to counteract air pollution effects by increasing the antioxidant enzyme activity (e.g., through a more rapid bone marrow response to PM) [117].

The null genotype for glutathione-S transferase M1 (GSTM1) had a modifying effect in the relationship between PM and AS, resulting in a more marked endothelial dysfunction (flow-mediated dilation) associated with PM exposure in T2D subjects [118]. The adhesion molecules have a role in AS since they are involved in the attraction and tethering of leukocytes to the blood vessels. The same polymorphism also modulates the association between PM and inflammation and endothelial dysfunction, as adhesion molecules, vascular cell adhesion molecule-1 (VCAM-1) and intercellular adhesion molecule-1 (ICAM-1) were particularly higher in subjects carrying the GSTM1 null gene [119].

Also, abnormalities in uric acid concentration have been found to be associated with PM, especially in people exposed to high pollution levels [120,121,122]. However, as many other antioxidants, uric acid at elevated levels may exert a pro-oxidizing effect instead of antioxidant actions, thus shifting by protection to potentially harmful [123]. Thus, further research is expected to better understand the role of uric acid increase related to air pollutants [124].

Higher PM_2.5_ has been recently found to induce a reduction of bilirubin (and biliverdin, molecules related to heme metabolism) in males; to note, bilirubin seems to represent a stronger risk factor for endothelial dysfunction and AS in men, thus the significance of these observed gender-related differences in the relationship with pollution merit further deepening [125,126].

Experimental data also showed that the activity of antioxidants enzymes (GPx together with SOD and catalase) is significantly reduced in the rat ischemic heart tissue exposed to PM_10_ [127]. Other experimental data (rats) showed that chronic exposure to O_3_ reduced cardiac function increased myocardial OS and inflammation in parallel to a reduction of heart SOD activity [128].

Thus, although more data are needed in this research scenario to better evaluate the complexity of the mechanisms involved and the OS-related effects induced by PM exposure, the overexpression of antioxidant enzymes could represent an effective preventive strategy to counteract damage induced by oxidant pollutants [129]. For example, inhaled glutathione seems to increase pulmonary glutathione, a fact which could represent a potential additive preventive tool to mitigate the adverse effects of pollution [130]. Moreover, recent experimental data (mice) suggest that aerobic exercise may act as a tool to both reduce OS and improve antioxidant capacity against negative effects related to PM exposure, a measure which seems particularly effective in older animals [131].

PM can directly generate reactive oxygen species (ROS), molecules that damage cells, or can indirectly cause ROS production [132]. This process, known as OS, can also be initiated by inhaling other toxic compounds present in air pollution [133]. OS is a state where higher levels of free radicals, ROS, are accumulated in different parts of the body, including the lungs, vascular bed and even at a local cellular/tissue level. Therefore, OS is a pathological condition that occurs whenever there is an imbalance between the production of ROS and the body’s ability to eliminate them, neutralize them or repair the damage caused by them, acting with antioxidant systems. When OS occurs, the disruption of redox signaling and excess ROS are suggested to increase adverse biological effects (lipid/protein/DNA oxidation and initiation of proinflammatory cascades) and consequently may alter cardiac and vascular function through the disruption of important redox-sensitive signaling pathways, the depletion of vasodilators and antioxidants, the perturbation of cellular mechanisms and the oxidation of proteins and lipids [134,135].

Exposure to PM_2.5_ has been associated to a variety of biomarkers; pro-inflammatory cytokines (e.g., interleukin-6 (IL-6), tumor necrosis factor (TNF)), acute phase proteins (e.g., C-reactive protein), vasoactive parameters (e.g., endothelin 1, NO), OS biomarkers (e.g., malondialdehyde MDA, oxidized low-density lipoprotein (Ox-LDL) and biomarkers of DNA oxidative modification, isoprostanes, protein carbonyls and nitrotyrosine, homocysteine) as well as different antioxidants in healthy subjects or in CV patients. A study including 40 healthy college students measured the plasma levels of Ox-LDL, highlighting that certain PM_2.5_ chemical constituents/pollution sources were more closely associated with changes in biomarkers of OS associated with AS [136]. Results showed that PM_2.5_ iron and nickel, as well as PM_2.5_ from traffic emissions and coal combustion, were positively associated with Ox-LDL, and PM_2.5_ calcium was associated with an increase in soluble CD36 [136]. Accordingly, air pollutants (PM_2.5_ and constituents, SO_2_, CO, NO_2_ and O_3_) measured during the 2008 Beijing Olympics resulted associated with acute changes in biomarkers of pulmonary and systemic inflammation, OS, and hemostasis and CV physiology biomarkers (heart rate and systolic blood pressure) in healthy, young adults [137].

The ROS can oxidize low-density lipoprotein (LDL), a key factor causing the onset and development of the atherosclerotic plaque, until the plaque can become unstable, resulting in the rupture, leading to acute disease manifestations [138,139]. Specifically, Ox-LDL are recognized by scavenger receptors (CD36) on the macrophages, which engulf Ox-LDL, resulting in foam cell formation, in turn enhancing OS and vascular inflammation and AS progression [140,141]. The scavenger receptor CD36 contributes to the inflammation associated with T2D, AS and thrombosis through the promotion of OS and its signaling to stress kinases [142]. Different studies examined the relationship between air pollution exposure and Ox-LDL levels, finding positive associations in occupationally exposed subjects, patients with T2D, and children [143,144,145]. Occupational exposure to vehicle emissions (PM_10_, PM_2.5_ and PAHs) revealed greater levels of several OS biomarkers (such as 8-oxo-2′-deoxyguanosine-a marker of DNA oxidative modification, 15-F(2t)-isoprostane in the urine, and blood levels of protein carbonyls and nitrotyrosine and lower levels of blood antioxidants) compared to controls [146,147]. A similar study, performed on taxi drivers, reported a positive correlation between increased urinary 1-hydroxypyrene, a biomarker of PAH exposure, and Ox-LDL and homocysteine [148]. Exposure to PM_2.5_ has been shown to cause oxidative and methylated DNA damage (8-hydroxy-2-deoxyguanosine and N7-methylguanine) in young subjects [149]. Elevated levels of LDL also have been found associated with traffic-related air pollution in Shanghai with consequent increased blood pressure, and homeostatic model assessment for insulin resistance (HOMA-IR, indicator of insulin resistance) and decreased antioxidant capacity (low levels of NO, SOD and total antioxidant capacity) [150]. Moreover, a recent meta-analysis found significant short-term associations of PM with TNF-α and fibrinogen (the percent change of a 10 μg/m^3^ PM_2.5_ increase on TNF-α and fibrinogen was 3.51%, 95% CI: 1.21–5.81%; 0.54%, 95% CI: 0.21–0.86%, respectively, and between PM_10_ and fibrinogen the percent change resulted 0.17%, 95% CI: 0.04–0.29%) [151].

The oxidative potential of PM_2.5_ has been found to be related to the risk of AMI: particles with the highest oxidative potential were associated with approximately an 8% increase in hospital admissions for AMI [152]. OS, measured as increased blood MDA levels, has been found in patients with acute coronary events associated with black carbon exposure [153]. Another study found that long-term ambient PM_2.5_ exposures were significantly associated with levels of multiple extracellular vesicle-encapsulated microRNAs in human serum [154]. In fact, pathway analysis on these extracellular vesicle-encapsulated microRNAs associated with PM_2.5_ identified several key related pathways promoting OS, inflammation and AS.

Experimental studies have substantially confirmed the effect of PM exposure on CV risk throughout the induction of oxidative/inflammatory responses. Some studies investigated the molecular pathway through which OS acts following exposure to different types of PM. Thickness of coronary arteries, accompanied by angiotensin pathways upregulation and a decrease of heme oxygenase-1 levels, was observed in healthy rats after PM inhalation [155]. A study revealed that the exposure to diesel exhaust (DE) in ApoE-/- mice increased plaque lipid content, foam cell formation and smooth muscle and the expression of plaque OS biomarkers such as inducible NO synthase (iNOS; inflammatory-related enzyme involved in NO production), CD36 and 3-nitrotyrosine [156] and was associated with impaired HDL antioxidant capacity [157]. A DE particle instillation study used a dose representing the upper range a person could be daily exposed to in a highly polluted city. Results showed an increment of plaque size, number, lipid rich area and frequency of buried fibrous caps in ApoE-/- correlating with lung inflammation (plaques per section of artery and buried fibrous layers) and antioxidant gene expression in the liver (NF-E2-related factor-2, NAD(P)H-quinone oxidoreductase 1 and heme oxygenase-1), indicating a response to systemic pro-oxidative effects [158]. Inhalation exposures to environmental air pollutants from vehicular sources (diesel PM and gasoline exhaust) in ApoE-/- mice resulted in vascular OS mediated by LOX-1 (main Ox-LDL receptor of endothelial cells), expression of MMP-9 (a predictor of atherosclerotic plaque instability) and ET-1 (the most potent vasoconstrictor) and monocyte/macrophage infiltration, associated with progression of AS, atherosclerotic plaque rupture and therefore AMI [159,160]. Direct addition of DE particles to cultured cardiomyocytes reduces contractile function, an effect that could be partially prevented by antioxidants [161]. Similarly, PM_2.5_, which is able to reduce antioxidant capacity, also causes a decrease in cardiomyocyte contractility upon direct exposure [162].

In LDLR-/- mice, UFP exposures have been shown to trigger reduced HDL antioxidant capacity, pro-atherogenic lipid metabolism and a greater atherosclerotic lesion [163,164]. Subacute PM_2.5_ exposure caused insulin resistance through OS, inflammation and the inhibition of the Phosphoinositide 3-kinase-Protein Kinase B (PI3K-AKT) signaling pathway, as evidenced by increased glucose levels in cell supernatants, and elevated insulin levels in parallel to impaired intraperitoneal glucose tolerance test in mice; PM_2.5_ increased OS (ROS, cytochrome P450 2E1 and MDA), and reduced SOD 1/2 and silent information regulator 1. Cytokines (IL-6 and TNF-α) were upregulated, while the PI3K-AKT signaling pathway was inhibited (decreased phosphorylation of PI3K/AKT in HepG2 cells) [165].

However, the effect of other pollutants beyond PM must also be considered: for example, CO exacerbated myocardial injury and depletion of antioxidants in the heart of a rat model of AMI [166]. The CO levels used were at the upper ranges of ambient levels in heavily polluted urban cities (30 ppm), spiked with peaks representative of that very close proximity to vehicle exhaust (100 ppm) [166]. Another work highlighted the involvement of the overexpression of iNOS mediating the higher sensitivity of the myocardium to ischemic events during a simulated urban CO air pollution exposure to daily non-toxic levels (30–100 ppm CO for 4 weeks) [167]. After chronic O_3_ exposure (0.8 ppm, 8 h/day for 28 and 56 days), cardiac function decreased in O_3_-exposed ischemia/reperfusion rats’ hearts [168]. The authors associated this enhanced sensitivity to ischemia/reperfusion injury with increased myocardial TNF-alpha levels and lipid peroxidation and decreased myocardial activities of SOD and interleukin 10 (IL-10) [168].

## 6. Metabolomics and Lipidomics Study

Metabolomic and lipidomic are promising tools to identify air pollution-related biomarkers by identifying a lot of metabolic and lipidomic features associated with exogenous exposures and endogenous processes [169]. Thus, focusing on changes in air pollution-related metabolites, these techniques could help in preventing air pollution-induced cardiometabolic risk.

Recently, a metabolome-wide association study was conducted by high-resolution metabolomics, an innovative analytical platform, in 1096 women, and annual average individual exposures to PM, NO_2_, O_3_, SO_2_ and CO were registered in the same year of blood draw. Metabolomics profiling showed that ninety-five metabolites were significantly associated with at least one air pollutant or mixture and related to pathways involved in the OS, energy metabolism, systemic inflammation, signal transduction, nucleic acid damage and repair. In particular, several amino acid pathways were significantly associated with exposure to PM_10_ and O_3_, including the urea cycle, tryptophan metabolism, methionine, cysteine, S-adenosylmethionine and taurine metabolism. Tryptophan metabolites were positively associated with PM_10_ exposure and negatively associated with CO exposure [170]. Using a global untargeted metabolomic approach in a longitudinal aging study among men (*n*  =  2280), several significant metabolites and metabolic pathways associated with long-term exposure to PM_2.5_, NO_2_ and temperature were identified in the blood samples. The results identify eight metabolic pathways perturbed by long-term exposure to PM_2.5_ and temperature: glycerophospholipid, glutathione, sphingolipid, beta-alanine, purine metabolism, biosynthesis of unsaturated fatty acids, propanoate and possibly taurine and hypotaurine metabolism. The perturbed pathways, such as glycerophospholipid metabolism and unsaturated fatty acids biosynthesis, were linked to OS since these molecules are the main components of biological membranes as well as downstream products from oxidation of the membranes, respectively [171]. In a prospective case-control study involving 1621 incident coronary heart disease cases and matched controls, 161 lipid species were evaluated using liquid chromatography-mass spectrometry in baseline fasting plasma. A panel of seven lipids was indicated as the best biomarker for the prediction of incident CVD: phosphatidylcholine 36:0a, sphingomyelin 41:1b, cholesteryl ester 18:2, sphingomyelin 34:0, phosphatidylethanolamine 36:4a, lysophosphatidylcholine 18:0 and 20:3 sphingolipids and phospholipids showed the most extensive associations with CAD risk [172]. Recently, in 244,842 participants from the UK Biobank, the metabolic signatures associated with exposure to ambient air pollution were investigated, to explore the link with metabolic dysfunction-associated steatotic liver disease (MASLD); 87, 65, 76 and 71 metabolites were found as metabolic signatures of PM_2.5_, PM_10_, NO_2_ and NOx, respectively. Metabolites related to metabolic signatures were associated with several metabolic categories, such as lipids, amino acids, lipoproteins, fatty acids and metabolites related to inflammation. The same study demonstrated that higher levels of PM_2.5_, PM_10_, NO_2_ and NOx were associated with an increased incidence of MASLD and that mixed exposure to different air pollutants also increased the risk [173].

## 7. Particulate Matter, Genomic Instability, Epigenetic Changes and Mitochondrial Dysfunction

Exposure to PM_2.5_ accelerates cellular and molecular detrimental effects, affecting genomic instability, telomere attrition, epigenetic changes and mitochondrial dysfunction. Toxicological mechanisms of PM_2.5_ in cells interfere with correct cell proliferation and alter metabolic and gene functions, eliciting inflammatory response and ROS production, contributing to the pathogenesis of various diseases, such as CV, neurodegenerative and musculoskeletal disorders [174]. The genome’s integrity is ensured by telomeres, repeated DNA sequences that act as a cap to preserve the end of chromosomes from being recognized as double-strand breaks and from undergoing degradation [175]. At each cell division, telomeres shorten, and this process continues until the cell stops dividing and enters senescence and apoptosis. For this property, telomere length (TL) is considered a biological marker of aging. Because of their sequence (TTAGGG repeats), due to the high number of guanines, telomeres are susceptible to ROS attack [176]. Many epidemiological studies have assessed the susceptibility of telomeres to shortening, both after short- or long-term exposure to PM_2.5_, and have highlighted an association of TL erosion with age-associated conditions such as CV and neurological diseases [177,178]. In a meta-analysis assessing the impact of outdoor PM_2.5_ exposure on TL, it was shown that long-term exposure had a greater impact on telomere attrition, principally by OS mechanisms. Oxidative damage at telomeres triggers the activation of DNA repair enzymatic machinery, which further amplifies the accumulation of telomere attrition leading to senescence and apoptotic processes [179].

Abnormal DNA methylation patterns are among the primary causes of alterations induced by PM_2.5_ exposure. DNA methylation is an important epigenetic feature of DNA that plays a critical role in gene regulation since it is a natural process that suppresses gene expression via the addition of methyl groups. There is growing evidence that PM interferes with global [180] and gene-specific methylation [181]. Several studies on prolonged exposure to PM showed a hypomethylation pattern of transposable repeated elements and proinflammatory genes [182,183]. Recently, controlled studies on human exposure to concentrated ambient particles provided the opportunity to experimentally observe the effects on blood pressure of rapid PM-induced DNA hypomethylation of Alu repeated elements and proinflammatory toll-like receptor-4, both linked to blood pressure and hypertension [184].

Many epidemiological and observational studies highlighted that mitochondria are extremely sensitive targets of PM_2.5_, which can severely damage the morphology, function and DNA of these organelles, contributing to adverse outcomes in various pathological conditions [185]. Mitochondria serve as central players of cellular metabolism, bioenergetics and OS [186], and, upon exposure to PM_2.5_, they undergo important structural and functional alterations, impairment of respiratory chain activity and dysregulation of quality control mechanisms. The large amounts of ROS released from damaged mitochondria, in turn, exacerbate the intracellular redox imbalance, further compromising mitochondrial stability, thus acting as both a cause and a consequence of oxidative and inflammatory states [187]. In particular, human vascular endothelial cells are susceptible to PM that induces disturbances in mitochondrial homeostasis: when PM damages mitochondria, adenosine triphosphate (ATP) production is impaired, compromising muscle contractility and causing early cell death [188]. The heart functions properly if it is provided with a constant and appropriate amount of energy in the form of ATP that is generated through β-oxidation during the Krebs cycle, which is therefore an important pathway for energy metabolism and is regulated by glucose and insulin [189]. PM has been observed to influence energy metabolism, reducing ATP production, and may therefore contribute to myocardial damage and consequently induce AMI [190]. Epidemiological studies have suggested that exposure to air pollutants may disturb glucose–insulin homeostasis [191], and short-term exposure in humans confirms that exposure to PM causes disturbances in Krebs cycles and glycolysis [192]. Disturbing the Krebs cycle may therefore lead to imbalances in cardiac energy metabolism, which we now know contributes to the onset of many CV clinical manifestations, including AMI [193].

## 8. General Prevention Strategies

Clearly, the strategies useful to directly reduce pollution, especially the sources of PM_2.5_, including traffic emissions, the use of fossil fuels for energy production and the burning of biomass, are critical targets to minimize adverse health effects and exposure of more vulnerable subjects [194]. In this context, different data suggest that targeted measures to prevent pollution and reduce PM_2.5_ can reliably decrease the risk of AS and CVD and reduce blood levels of biomarkers of OS and inflammation [195,196].

Overall, available evidence indicates that there is no “unharmful” level of PM exposure. Moreover, although WHO data show that the majority of the global population lives in areas that exceed WHO guideline air quality limits, both susceptible individuals and healthy subjects are not fully aware of their risk, which renders a great challenge to fully protect public health [197].

Public strategies, the most important are pollution regulations (establish air quality standards), but also urban planning (e.g., green areas and parks) and educational campaigns (to improve public education and awareness) or improve public transports (e.g., decreasing emissions and encouraging physical activity) are simple but essential interventions to reduce exposure risk. General simple not demanding interventions, such as closing windows (when elevated ambient pollution levels)/opening windows (to ventilate indoor environments when outdoor pollution is low), the use of air purifiers and effective dustproof masks may be effective preventive measures to reduce personal exposure. Moreover, additive tools (e.g., air quality warning systems to notify daily pollution levels, wearable devices, new exposure models, geospatial assessment) are under study.

In the context of CV settings, PM exposure should be considered as a major modifiable risk factor, to be considered in the promotion of a healthy lifestyle and behaviors (together with a healthy diet, physical activity, maintenance of a normal blood pressure/lipid profile/glycemia values and smoking habit cessation), the control of which in turns reduces the susceptibility to CV events attributed to air pollution exposure. The identification of more vulnerable subjects for exposure risk by physicians may be more accurate considering traditional risk factors and pre-existing disease, but also additive key information such as socioeconomic status, lifestyle and work conditions, which can have an important role.

## 9. Antioxidant Strategies

### 9.1. Antioxidant Nutrients and Healthy Dietary Habits

Beyond reductions in air pollution, one further option to counteract the adverse effects of PM on health may be to increase the intake of antioxidants and/or exploit some antioxidant drug properties, found to be effective in reducing air-pollution induced OS and inflammation and as such able to counteract air-pollution negative repercussions on health [198]. Thus, in view of the consistent data highlighting the role of OS in the effects of air pollution on health, there is substantial evidence on the potential use of antioxidant supplementation (e.g., vitamins B12, C, D and E or omega-3 polyunsaturated fatty acids—omega-3 PUFAs), to improve the capacity to counteract adverse pollutant consequence, through OS reduction [199]. Accordingly, a relationship has been hypothesized between different micronutrients, including vitamin B12 and folate and heart rate variability (HRV: the variation of time between consecutive heartbeats, biomarker associated with CV pathophysiology); in this context, although number and heterogeneity between studies did not allow a definitive and clear conclusion for majority of these molecules, increasing findings seem to confirm the association between vitamin D and B12 with reduced HRV [200].

In vitro data showed that vitamin E and omega-3 fatty acids significantly reduced PM_2.5_-induced inflammation and OS (MDA, IL-6) and TNF-α decreased in supernatant and ROS decreased in cytoplasm, while SOD activity increased [201]. In rats, the combined treatment with vitamin E and omega-3 PUFA prevents the PM_2.5_-induced CV injury through alleviating inflammation (TNF-α, interleukin 1β (IL-1β), IL-6) and OS (anti-oxidative activity) [202].

Direct cytotoxicity or apoptotic effects of various PM types in cardiomyocytes could be inhibited by compounds with antioxidant properties (e.g., N-acetylcysteine NAC, an antioxidant precursor of cysteine and glutathione, used to treat paracetamol overdose and dietary supplement or dimethylthiourea, a scavenger of hydroxyl radicals and hydrogen peroxide) [203,204]. In H9C2 cardiomyocytes, PM_2.5_ exposure induces different changes (reduction in cell viability, death, ROS, and increased expression of caspase-3, fatty acid binding protein 3 and IL-6, as well as upregulation of lysophosphatidylcholines and dysregulation of amino acids and other acids and derivatives) that can be improved by vitamin C treatment [205].

Interestingly, vitamin B supplementation attenuated the epigenetic changes (methylation changes in genes involved in mitochondrial oxidative energy metabolism) induced by PM_2.5_ exposure in humans [206]. Other experimental data showed that vitamin B may improve PM_2.5_-induced kidney injury by counteracting endoplasmic reticulum stress and OS [207].

Omega-3 PUFA reduces adverse CV effects of short-term exposure to air pollution, in terms of lipid profile and biomarkers of OS, inflammation, coagulation and endothelial function in healthy middle-aged subjects [208,209]. Fish oil also improved PM_2.5_-induced lung toxicity and systemic inflammation in rats (evidenced by increased levels of total proteins, lactate dehydrogenase, 8-epi-Prostaglandin F2α, IL-1β and TNF-α, and increased infiltration of inflammatory cells, decreased SOD in the bronchoalveolar lavage fluids, and elevated blood C reactive protein and IL-6) [210]. Moreover, a relationship between short-term exposure to PM_2.5_, even at concentrations below the regulatory standard, and subclinical CV biomarker changes (total cholesterol, von Willebrand factor, tissue plasminogen activator, D-dimer and HRV) was observed in healthy adults, which was relieved by omega-3 PUFA consumption [211]. In this context, administration of vanillic acid (phenolic acid and an oxidized vanillin form) in an experimental model of ischemia/reperfusion isolated rat heart exposed to PM_10_ resulted in cardioprotection, as evidenced by effects on hemodynamic parameters, OS and antioxidant enzymes, and endothelial NO synthase (eNOS) and iNOS mRNA expression levels [212].

Recently, air pollutant exposure, low vitamin D status and smoking habits were demonstrated to confer a high risk of hypercholesterolemia (*n* = 28,134 Korean adults) [213]. Experimental data evidenced the protective role of vitamin D receptor against PM_2.5_-induced injury in the kidney, as vitamin D receptor activation restores mitochondrial calcium balance and reduces OS, downregulating mitochondrial calcium uniporter expression and improving renal function [214].

A healthy diet can represent a simple tool to increase antioxidant intake. In particular, the Mediterranean diet (MD: rich in fruits and vegetables, olive oil, oily fish and moderate alcohol consumption, e.g., antioxidant-rich red wine) appears beneficial to prevent and/or reduce air pollution-associated adverse health effects. A recent prospective study (*n* = 548,845 in the USA, follow-up period of 1995–2011) evidenced that MD reduced the CV mortality risk related to long-term exposure to air pollutants (PM_2.5_ and NO_2_) [215]. An inverse association between adherence to MD and exposure to PM_10_ with LINE-1 methylation suggested the possible beneficial role of a healthy diet to counteract the negative effect of PM_10_ exposure [216]. A recent review evaluated the effects of some healthy diets (MD, Dietary Approaches to Stop Hypertension DASH, and MD-DASH intervention for Neurodegenerative Delay), which promote a favorable OS and inflammation status, and positive changes in the composition of the human gut microbiota (mitigating adverse effects caused by pollutants on Alzheimer’s disease) [217].

According to available data, high consumption of flavonoids (polyphenolic metabolites contained in fruits, vegetables, tea, cocoa, wine, nuts, seeds, spices and other plant-based foods) with their antioxidant and anti-inflammatory properties, consistently resulted in counteracting the CV damage caused by different pollutants (e.g., P_2.5_, PM_10_, NO_2_, O_3_ and SO_2_), which are instead linked to increased risks of hypertension, stroke, AMI, atrial fibrillation and HF [218]. Hydroxytyrosol (a polyphenol contained in extra virgin olive oil) reduces hepatic insulin resistance (through inhibition of nuclear factor kappa-light-chain-enhancer of activated B cell (NF-κB) activation derived from OS induced by PM_2.5_) [219]. Curcumin, resveratrol and gallic acid prevented PM_2.5_-induced migration and cytokine secretion via blocking the ROS-dependent NF-κB signaling pathway in vascular smooth muscle cells [220]. Moreover, different results evidenced how resveratrol can reduce the effects of PM_2.5_ exposure in different districts by antagonizing OS and inflammatory responses [221,222,223].

### 9.2. Probiotics

Recently, probiotic intervention has been the object of interest in view of the capacity to decrease inflammation processes induced by pollutants, especially regarding pulmonary effects. In fact, there is evidence of beneficial effects of the probiotic *Lactiplantibacillus plantarum* in PM-associated pulmonary inflammation [224]. Moreover, experimental data showed that probiotic administration improves lung function, reducing proinflammatory cytokines expression (TNF-α, IL-6, IL-1β, interleukin 17A) while increasing anti-inflammatory mediators (IL-10, transforming growth factor-β) [225]. Gut microbial abundance, which can be modulated by diet and probiotics supplementation, has been negatively associated with asthma incidence derived from PM_2.5_ exposure [226].

In addition, there are data on the beneficial effects of probiotic administration on ischemic CV damage, where probiotic administration was cardioprotective on myocardial ischemic injury through reduction of inflammation and OS [227,228,229]. Experimental data suggest that probiotics reduce the AMI size and post-infarction cardiac hypertrophy and HF, whereas very recent data confirmed that gut microbiome modulation (L. johnsonii) improves the cardiac function post-AMI [230,231,232].

### 9.3. Cardiovascular Drugs

#### 9.3.1. Beta-Blockers

There is a complex relationship between OS, the autonomic system (on which beta-blockers act) and air pollution, targeting the CV system. When the daily variations in ambient particulate air pollution have been investigated in association with increased risk of ST-segment depression during the exercise test in CAD patients, the associations of PM_2.5_ and gaseous pollutants (NO_2_ and CO) were stronger among patients who did not use β-blockers, likely in view of the protective effect of β-blockers on ischemia, which may reflect autonomic regulation (likely through OS, but that unfortunately was not specifically evaluated in this study) [233].

Afterwards, experimental data indicated the changes in HRV of rats, exposed to intratracheal instillation of urban air particles (UAP, 750 µg) or to inhalation of concentrated ambient particles (mass concentration 700 ± 180 µg/m^3^) for 5 h, can be inhibited by NAC (50 mg/kg 1 h prior to UAP), while the cardiac OS induced by pollution can be prevented by beta-blocker (5 mg/kg atenolol immediately before concentrated ambient particles exposure) [234]. Moreover, pulmonary rat exposure to DE particulate increases blood pressure, arrhythmia and reperfusion injury with adverse effect on the myocardium (increased myocardial oxidant radical production, tissue apoptosis and necrosis), whereas metoprolol (10 mg/kg) prevents myocardial OS and reperfusion injury [235]. Instead, propranolol inhibits PM-induced IL-6 release from murine alveolar macrophages, while β2-adrenergic receptor (β2AR-albuterol) increases IL-6 release, mitochondrial ROS and adenylyl cyclase activity, finally modulating the coagulable status and risk of thrombotic CV events [236].

#### 9.3.2. Statins

In a large case-control study (1.2 million adults aged ≥66 years in Canada, 2000–2018) the associations of chronic exposure to PM_2.5_ with CV mortality were stronger among non-statin users compared to users, suggesting that these drugs may act as significant modifiers in the relationship between pollution and CV death [237].

The anti-inflammatory and antioxidant properties of statins seem one of the main mechanisms involved in the pollution defense, because among GSTM1-null subjects (GST are enzymes involved in the metabolism of ROS and xenobiotics), enrolled in the Normative Aging Study (*n* = 497 individuals), the use of statins eliminated the adverse effect of PM_2.5_ on high-frequency component of HRV [238].

Accordingly, in PM_2.5_ rat exposure models, atorvastatin significantly improved lipid profile, OS and inflammatory-related biomarkers (MDA, SOD, Ox-LDL, high sensitivity C reactive protein), cytokines (IL-6, TNF-α) and blood pressure, reversing the effects caused by pollutant exposure [239,240]. The modulatory effect of statins in the relationship between pollution and inflammatory and endothelial function biomarkers was also observed in humans [241,242]. Nonetheless, the association of PM_2.5_ with incident AMI seems not attenuated by statin therapy in another study; however, as authors declare, this information was available only at baseline, with possible changes in therapy during the follow-up period that could have affected the role of statins on the association between PM_2.5_ and incident AMI [243]. In this context, recent data reported that statin use was associated with a significantly lower risk of stroke among the elderly with high and low or moderate levels of exposure to PM_10_ and PM_2.5_ [244].

#### 9.3.3. Angiotensin Converting Enzyme Inhibitors and Angiotensin Receptor Blockers

Experimental data focused on rats treated with benazepril (ACE inhibitor) or valsartan (an angiotensin receptor blocker ARB) before exposure to fine PM aerosols (5 h, fine PM mass concentration: 440 +/− 80 µg/m^3^) or filtered air; benazepril reduced, while ARB increased angiotensin levels; both drugs improved heart OS (thiobarbituric acid reactive substances) and ECG alterations (shortening of the T-end to T-peak interval), suggesting that PM-related acute cardiac events involved the renin–angiotensin system and can be modulated by ACE inhibitors and ARB [245].

Moreover, fine dust particles induced premature senescence-associated endothelial dysfunction in endothelial cells isolated from porcine coronary arteries. Specifically, fine dust increased senescence-associated beta-galactosidase activity, causing cell cycle arrest and OS, whereas eNOS expression was downregulated and platelet aggregation increased. Angiotensin II receptor type 1 (AT1) antagonist prevented fine dust-induced senescence-associated beta-galactosidase activity, increased cell proliferation and eNOS expression, and improved endothelial function, suggesting the involvement of the local angiotensin system in these events [246].

## 10. Final Remarks: Points to Solve and Future Directions

There are many key points in the assessment of the relationship between PM and health still to solve (Table 1).

### 10.1. Individual Issues and Biomarkers

Although no one questions the adverse health consequences of PM in view of the number of findings confirming this relationship, it remains challenging to establish an unambiguous causal relationship between PM and its components with health effects. There are people who live in the same environment but are differentially affected by the PM, due to the fact that many modifiers of an individual’s response to PM effects exist. These factors surely include subject movements that influence individual exposure, socioeconomic parameters, the nutritional status, antioxidant supplementation, lifestyle habits adopted and outdoor activities, presence and severity of preexisting chronic diseases and risk factors as well as genetic background (especially candidate genes having specific role in antioxidant activities; e.g., the presence of polymorphisms that change the activity of the detoxification enzymes GST and quinone oxido-reductase-NQO1 may contribute to heart disease as well as to pollution adverse effects and potentially modulate their relationship and the final effects on health) [247,248]. Additional difficulties exist in the demonstration of the connection between PM and health, including difficulties in estimating emissions under all possible conditions and the individual exposure time. Variations in research populations and presence of confounding factors in observational studies that assess air pollution effects on health, such as lifestyle habits (e.g., diet, smoking habit, outdoor activities), socio-economic status, or preexisting risk factors (e.g., dyslipidemia, hypertension, diabetes) or diseases (e.g., CAD), can affect the final results. All these characteristics, which can vary greatly between subjects, affect individual susceptibility to pollution and the biological effects and health repercussions on each individual.

Several oxidative and inflammatory-related biomarkers have been measured and associated with PM; abnormalities in some key parameters related to PM evidence the importance of this relationship in terms of health effects. Nonetheless, further research is needed to identify the role of new biomarkers with innovative techniques (e.g., omics) which may reveal additive mechanistic links between air pollution and health, to fight even more effectively adverse pollution effects with reliable preventive and interventional tools.

### 10.2. Exposure Misclassification

Exposure assessment is a key determinant in all studies of environmental pollution effects, but several sources of exposure misclassification exist. Exposures are often estimated using a fixed monitoring point or the home locations of individual subjects, without considering residence-based and mobility-based exposures and their impact on health (e.g., which can occur due to residential changes among a study population over follow-up, i.e., residential mobility). Case-control studies often use questionnaires to collect information on exposure, but errors due to the use of questionnaires may be significant in terms of exposure misclassification.

Other methodological aspects may affect exposure assessment, which may often be characterized by using only one or a few blood samples, resulting also in this case in significant exposure misclassification. Moreover, variations of air pollution levels over time can impact exposure levels, and the use of national-scale models may not reflect local changes in air pollution exposure.

### 10.3. Sampling Methodology and Variability in Particulate Matter Measurement

Sampling and analysis instrumentation in the context of PM samplers may greatly differ depending on the specific application. The sampling methods employed by national environmental protection agencies are aligned with their respective National Ambient Air Quality Standards (NAAQS), but they may differ across countries in aspects such as aspiration efficiency, cutoff calibration, flow rate and flow measurement and control systems [249]. Beyond the stationary samplers used by national monitoring networks, a range of specialized instruments is also utilized, including indoor air quality monitors, mobile sampling units and personal dosimeters.

While ground-based measurements remain essential for accurately assessing PM concentrations at specific locations, they are inherently limited in spatial coverage. To overcome this limitation, satellite observations offer a valuable complementary tool for monitoring PM on a broader scale. Remote sensing platforms can provide near-global coverage and high temporal frequency, enabling the detection and tracking of large-scale aerosol events such as desert dust outbreaks, volcanic ash plumes and smoke from forest fires. By integrating satellite data with ground-based networks through data assimilation or statistical modeling approaches, it becomes possible to improve spatial estimates of PM concentrations, enhance early warning systems and support air quality forecasting and mitigation strategies at regional and global scales [250].

PM represents a complex and challenging system to study, being heterogeneous in composition. In fact, PM differs greatly in its chemical and physical characteristics, including size and distribution, composition and concentration, toxicity, emission sources, formation and transformation mechanisms, and diffusion patterns, which are influenced by geographical location and meteorological conditions (e.g., transportation and removal rate of atmospheric PM) until extreme events such as volcanic eruptions and dust storms. In particular, several weather-related factors influence the variability of PM measurements. These include temperature (low temperatures are associated with higher PM levels), solar radiation (higher radiation is typically associated with lower PM_10_ concentrations), wind (e.g., low wind speeds tend to correlate with higher PM levels), relative humidity (high humidity is linked to increased PM concentrations), precipitation (which reduces PM levels through atmospheric cleansing) and atmospheric pressure (high pressure is generally associated with higher PM concentrations). The nature and intensity of emission sources also play a significant role in PM measurements. Moreover, studies are usually limited to a few main pollutants without considering global mixture effects, or gaseous components (NO_2_, SO_2_, O_3_), which can have their role on their own.

### 10.4. Antioxidant-Related Strategies

Antioxidant supplementation is generally harmless (at modest doses at least) and largely available at low cost. This strategy may be effective (seeing the importance of both inflammatory and OS pathways affected by a number of air pollutants) and widely applicable as a general intervention targeting all populations (overcoming both the great variable pollution composition and individual variability in susceptibility). However, vitamin supplementation has largely disappointed expectations in large-scale trials with CV endpoints. The action mechanisms of each antioxidant (be supplemented or contained in the diet) have to be better defined in terms of cellular and molecular pathways involved in the effects, as well as the type of contribution (e.g., directly targeting pollutant effects or indirect, as a more general advantage independent of pollutant exposure). Thus, at present there is no clear evidence to recommend antioxidant supplementation to counteract the adverse consequences of air pollution, also in subgroups of subjects more vulnerable to pollution (e.g., very young and elderly, smokers, subjects with pre-existing cardiorespiratory conditions, those living in very polluted areas) where these strategies may provide more benefits to health.

Future studies should investigate OS-related mechanistic mechanisms to better understand how PM causes adverse health effects, with particular focus on signaling mediator functioning (e.g., through innovative techniques as metabolomic and lipidomic analyses) and the role of genomic instability, epigenetic changes and mitochondrial dysfunction.

Gut microbiome-targeted nutritional intervention may represent an innovative prophylactic strategy to mitigate damage after acute ischemic cardiac events in general, but also potentially effective in relation to the pathophysiological molecular and cellular events (e.g., OS and inflammation) elicited by pollutant exposure, although their precise role in air pollution remains to be validated.

Some common CV drugs have been found effective as modifiers in the relationship between OS elicited by pollutants and CV pathophysiology and thus worthy of evaluation as potentially profitably exploitable additional pharmacological tools for the prevention and/or treatment of air pollution-associated CVD in vulnerable patient groups.

## 11. Conclusions

**Figure 3:**
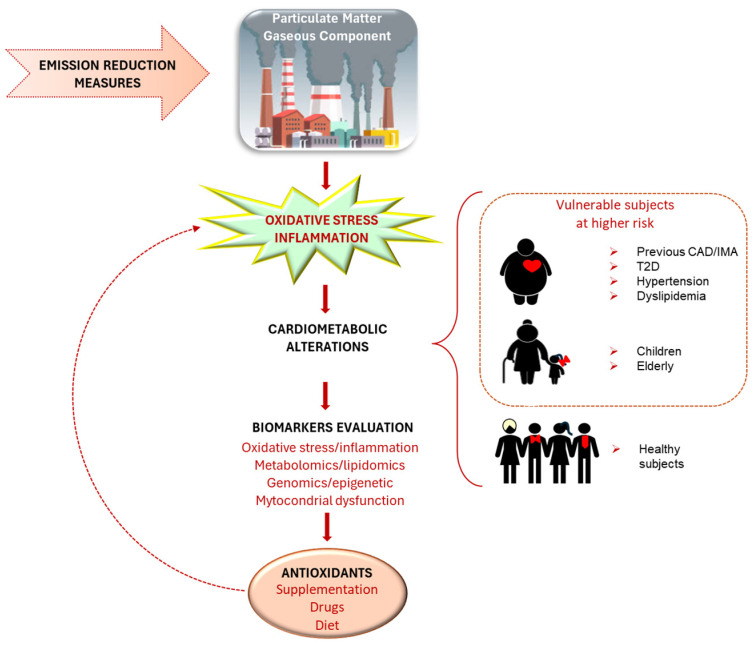
Pollution, oxidative stress/inflammation and related biological effects, which can be modulated by antioxidants, as potential additive tools beyond emission reduction measures, to counteract pollution’s detrimental effects on health.

Air pollution is by now a recognized risk for health, directly affecting the CV pathophysiology as well as because pollutants enter the bloodstream as a highway to distribute throughout the body to act on other organs; the sum of these events leads to an annual mortality rate in excess of a million people. OS, with its close relationship with inflammatory responses, represents a key determinant by which the actions of air pollution can be expanded to produce adverse responses in multiple organs, including the CV system (Figure 3). Future efforts will be necessary to address the accuracy and effectiveness of OS biomarkers and their association with pollutant characteristics (e.g., dimension, components and oxidative burden), but, above all, to assess the modulatory role of the antioxidant responses on these relationships.

Clearly, reducing the sources remains the main and more effective solution to reduce the burden of air pollution on health, although this strategy is not always easy to implement. In addition to this solution, experimental studies and clinical trials have demonstrated clear effectiveness of antioxidant compounds (dietary or drugs) in the prevention/reversal of pollutant adverse effects, confirming OS as a main determinant in these biological effects (Figure 3). However, whether an improvement of the OS and inflammatory status was associated with vitamin supplementation, a clear clinical benefit of supplementation of these nutrients in the relationship between air pollution and the cardiometabolic risk and disease remains to be definitively proved. Nonetheless, in view of the pivotal role of oxidative pathways in the air pollution consequences on health, if it is reasonable to think of the use of antioxidant compounds to improve adverse effects following exposure to pollutants in future, potentially beneficial also for other conditions driven by pollutant exposure and/or OS elevation, further data are needed to explain in detail the complex relationship between air pollution, OS and CV health.

## Figures and Tables

**Table 1:** Main critical points in the assessment of the relationship between PM, OS and health.

**PM-Related Factors**		
PM characteristics	different chemical and physical properties	-mass, -number -size, shape -surface area -reactivity -acidity -solubility -internal or surface positioning of chemicals on the particles
	heterogeneity in composition	-metals -salts -organic chemicals -biological materials
	varying mechanisms of formation	-nucleation process -condensation process -coagulation process -mechanical process
	anthropogenic and natural emission sources	-traffic, industrial activities, biomass burning, mineral desert dust, sea spray, biogenic emissions
		-geographical area asset
PM transport and deposition processes	diffusion, dilution and deposition patterns over time and space	-meteorological variables, as air temperature, humidity, wind and other parameters
Interaction with other pollutants	gaseous pollutants	NO_2_, SO_2_, O_3_
		no interaction vs. potential additivity, synergism or antagonism
Sampling methodology	differences between fixed monitoring points and mobile or individual monitoring assessment	-wearable device -sensors mounted on vehicles -geospatial assessment -fixed monitoring stations
	different monitoring instrumentation	different spatial and temporal resolution
**Individual Characteristics**		
Individual factors	anthropometric, genetic and social characteristics	-age -sex -genetic profile -socioeconomic status
	comorbidities and pre-existing diseases	-cardiovascular disease -respiratory diseases -presence of diabetes, dyslipidemia, hypertension
	behaviors	-diet -outdoor activity -occupation -exercise -smoking -mobility
**Modifying or Mitigating Factors**		
Antioxidant intake	choice of antioxidant	single compound vs. combination/cocktail
	mechanism of action	direct vs. indirect
	administration	-timing -appropriate dosage -duration of use
	use in the general population vs. targeted application in vulnerable groups	-children, -elderly, -smokers, -individuals with chronic conditions
Individual protectors	wearable or stationary technological devices	-face masks, -indoor air purifiers and filtration systems -air quality warning systems
	general measures	-air exchange
	health interventions	-regular medical screening in subjects at high-risk -lifestyle changes
Community interventions	environmental and policy strategies	-reducing source of pollution: policies to limit emissions -improvement of air quality assessment -urban planning -public health campaigns and education -improvement of public transports

## List of Abbreviations

ACE	Angiotensin-converting enzyme
AMI	Acute myocardial infarction
ARB	Angiotensin receptor blocker
AS	Atherosclerosis
ATP	Adenosine triphosphate
CAC	Coronary artery calcium
CI	Confidence interval
CV	Cardiovascular
CVD	Cardiovascular disease
Da	aerodynamic diameter
DE	Diesel exhaust
ECG	Electrocardiogram
GPx	Glutathione peroxidase
GPx-1	Glutathione peroxidase 1
GST	Glutathione-S transferase
GSTM1	Glutathione-S transferase M1
HDL	High-density lipoprotein
HF	Heart failure
HRV	Heart rate variability
IL-1β	Interleukin-1β
IL-6	Interleukin-6
IL-10	Interleukin-10
IMT	Intima media thickness
ICAM-1	Intercellular adhesion molecule-1
eNOS	Endothelial NO synthase
iNOS	Inducible NO synthase
LDL	Low-density lipoprotein
MASLD	Metabolic dysfunction-associated steatotic liver disease
MD	Mediterranean diet
MDA	Malondialdehyde
NF-κb	Nuclear factor kappa-light-chain-enhancer of activated B cell
NO	Nitric oxide
NO_2_	Nitrogen dioxide
OC	Organic compound
Omega-3 PUFA	Omega-3 polyunsaturated fatty acid
OS	Oxidative stress
Ox-LDL	Oxidized low-density lipoprotein
O_3_	Ozone
PAH	Polycyclic aromatic hydrocarbon
PI3K-AKT	Phosphoinositide 3-kinase-Protein Kinase B
PM	Particulate matter
PM_2.5_	Particulate matter with a diameter less than 2.5 µm
PM_10_	Particulate matter with a diameter less than 10 µm
ROS	Reactive oxygen species
SOD	Superoxide dismutase
SO_2_	Sulfur dioxide
TL	Telomere length
TNF	Tumor necrosis factor
TNF-α	Tumor necrosis factor α
UFP	Ultrafine particle
VCAM-1	Vascular cell adhesion molecule-1
WHO	World Health Organization
